# Delayed coronary artery occlusion after transcatheter aortic valve replacement and chimney stenting: a case report

**DOI:** 10.1186/s12872-021-02249-2

**Published:** 2021-09-17

**Authors:** Hui Li, Wenduo Zhang, Bo Xia, Fucheng Sun, Jiefu Yang, Huiping Zhang

**Affiliations:** 1 Fifth School of Clinical Medicine, Peking University, Beijing, People’s Republic of China; 2 Cardiology Department, Beijing Hospital, National Center of Gerontology, Institute of Geriatric Medicine, Chinese Academy of Medical Sciences, No. 1 DaHua Road, Dong Dan, Beijing, 100730 People’s Republic of China

**Keywords:** Coronary artery occlusion, Transcatheter aortic valve replacement, Chimney stenting

## Abstract

**Background:**

Delayed coronary artery occlusion (CAO) is a rare but fatal complication after transcatheter aortic valve replacement, chimney stenting is the standard technique for established CAO or impending CAO.

**Case presentation:**

We describe a female patient who developed non-ST elevation myocardial infarction after receiving transcatheter aortic valve replacement and chimney stenting 4 months prior. An angiogram revealed delayed coronary artery occlusion with a deformed stent, which was never reported. This patient was subsequently treated with a new chimney stent.

**Conclusions:**

For self-expanding valves, the coronary ostium is protected by chimney stenting, delayed coronary artery occlusion can occur and cause catastrophic complications.

**Supplementary Information:**

The online version contains supplementary material available at 10.1186/s12872-021-02249-2.

## Background

Coronary artery occlusion (CAO) is a rare but fatal complication after transcatheter aortic valve replacement (TAVR) that most frequently occurs within minutes after the TAVR procedure [1]. Delayed CAO is even rarer (0.22%), and the in-hospital death rate can be as high as 50% [2]. The usual preventive measures for CAO are premounted guidewires, balloons, or undeployed stents in the coronary artery, and chimney stenting is the standard technique for established CAO or impending CAO [3, 4]. Here, we report a case of delayed CAO with chimney stenting.

## Case presentation

**Fig. 1:**
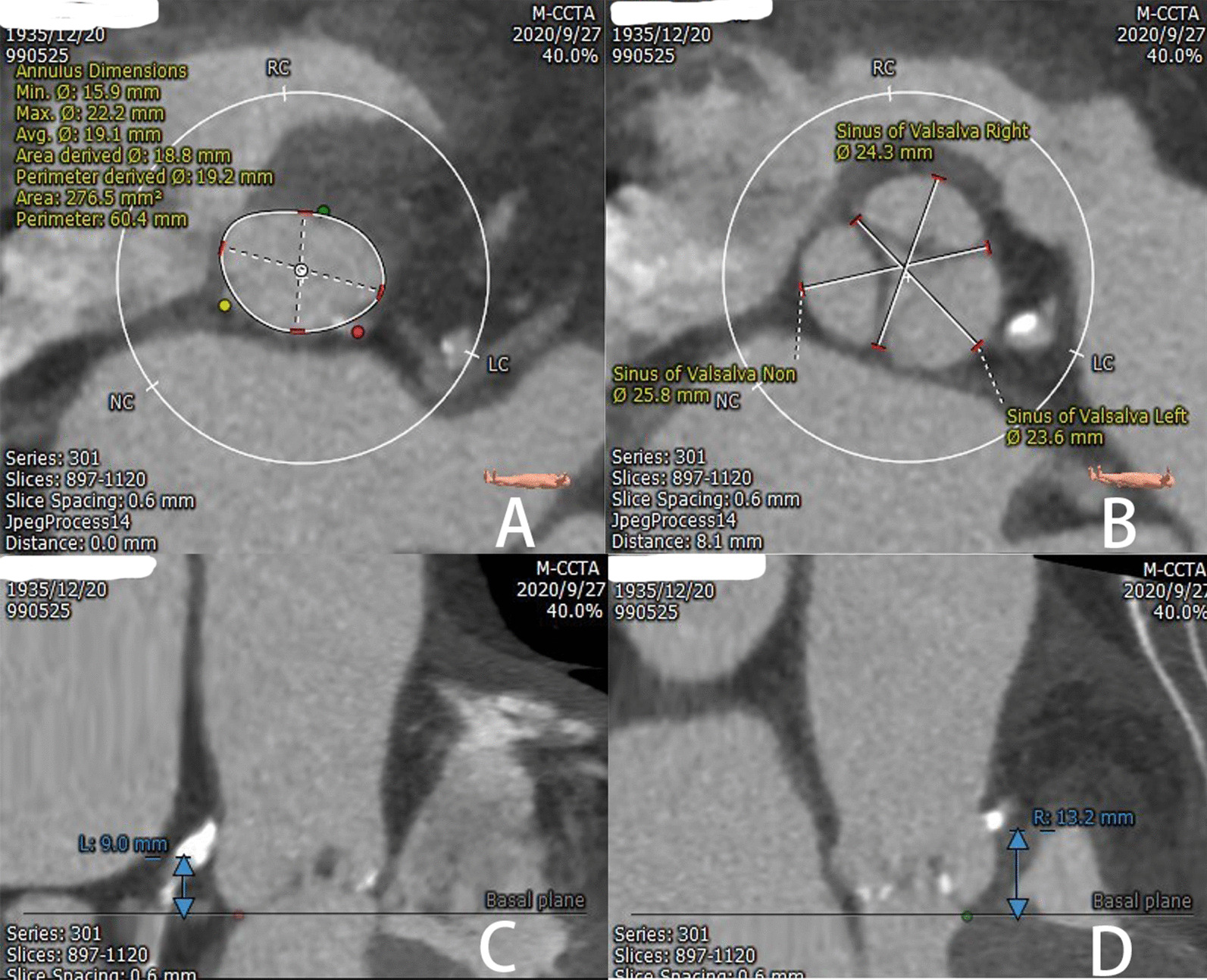
The measurements of CT scan. **a** Annulus diameters and perimeter; **b** Sinus of Valsalva depths; **c** Left coronary height; **d** Right coronary height

An 85-year-old female was admitted to our hospital for severe coronary heart disease and aortic valve stenosis. She was at high risk for cardiac surgery, and the calculation of the Society of Thoracic Surgeons (STS) score demonstrated the risk of mortality was 16.153%. Pre-procedure computed tomography (CT) scan revealed the annulus diameter was 19.1 mm (Fig. 1a), the left main (LM) ostium height was 9.0 mm (Fig. 1c), and the right coronary artery (RCA) ostium height was 13.2 mm (Fig. 1d).

**Fig. 2:**
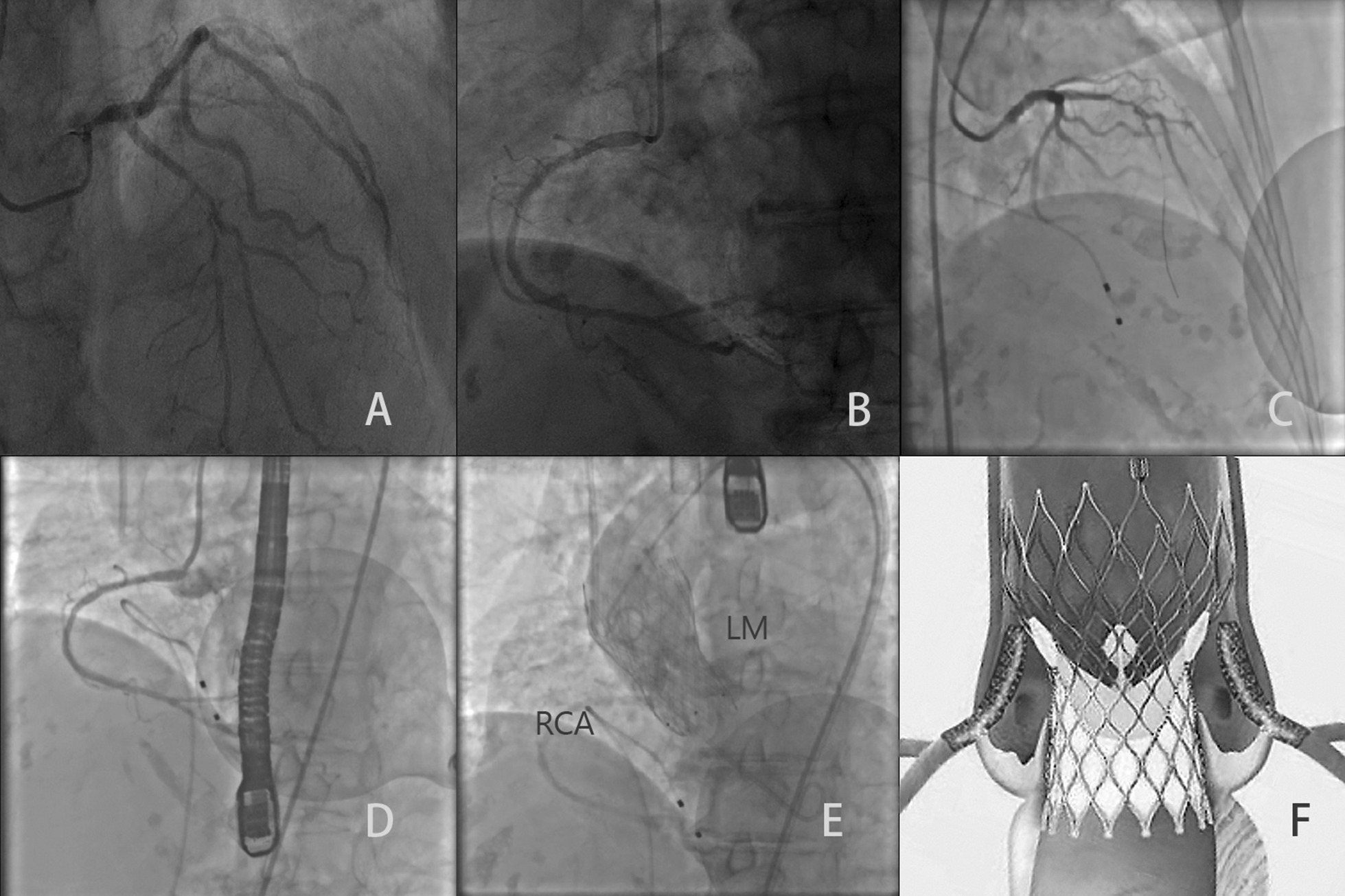
The coronary stenting and TAVR procedure. **a**, **b** Angiography of the left main and right coronary artery. **c**, **d** Stenting of the ostia of coronary arteries. **e** Aortic angiography after transcatheter aortic valve replacement. **f** Illustration of the position of the stents and the transcatheter heart valve

She had two drug-eluting stents deployed at the ostia of the LM and RCA, with approximately 1/3 of the stents protruding into the sinus of Valsalva (Fig. 2c, d). During the same procedure, a 26 mm Venus-A self-expanding valve (Venusmedtech, Hangzhou, China) was implanted. Kissing balloon inflation was not performed because of a lack of post dilatation of the transcatheter heart valve (THV). An aortic angiography demonstrated good perfusion of both coronary arteries (Fig. 2e). She had no clinical symptoms after the operation, was prescribed aspirin and clopidogrel as a dual antiplatelet therapy for the following 12 months. She was discharged 7 days after the procedure and was asymptomatic at the 3-month follow-up.

Four months later, the patient was admitted to the emergency department for non-ST elevation myocardial infarction. She developed epigastric discomfort for 2 h, and the electrocardiogram showed a transient ST-segment elevation in the avR lead and extensive ST-segment depression in the chest lead, with troponin I up to 16 ng/ml.

**Fig. 3:**
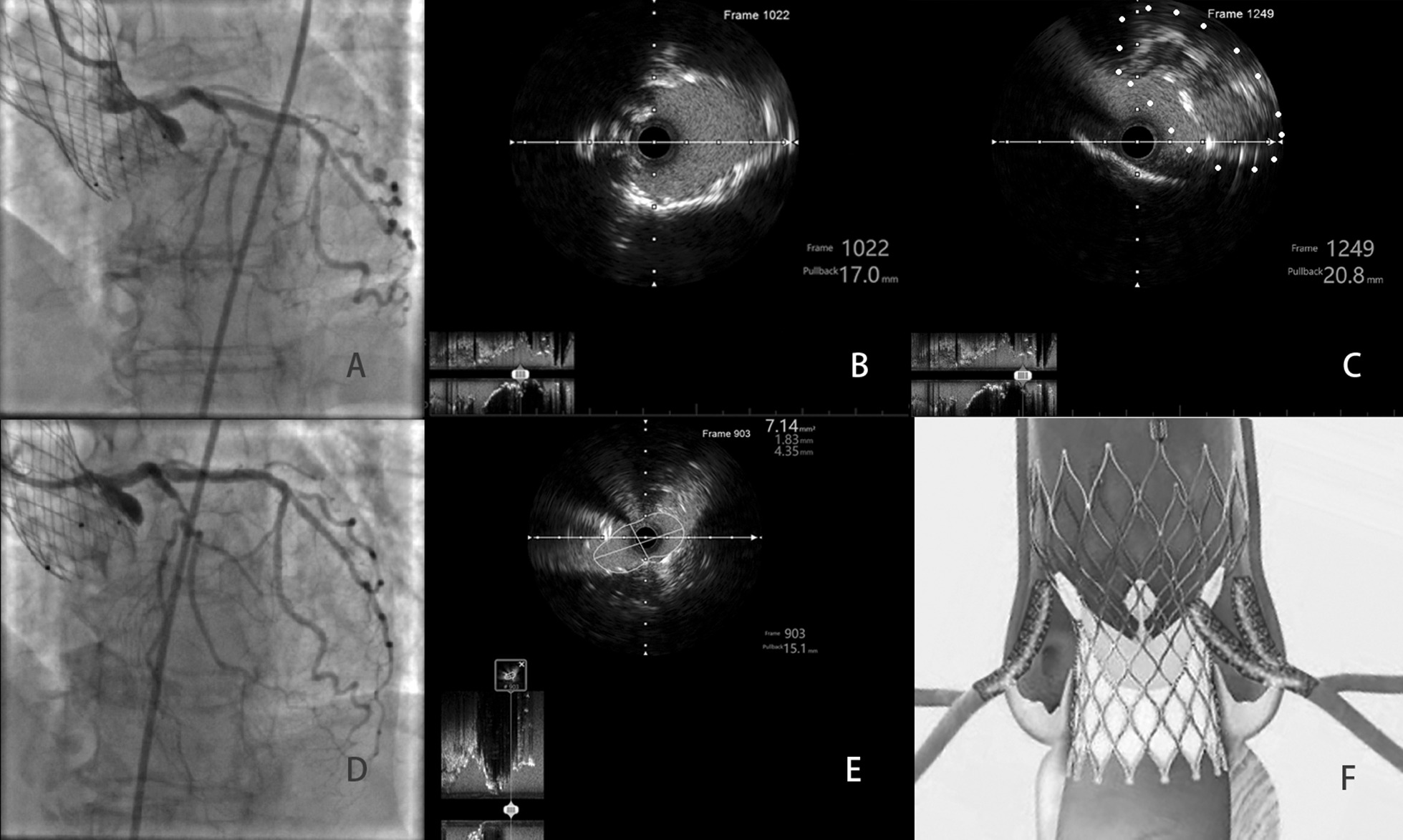
Re-intervention procedure and intravascular ultrasound (IVUS) images. **a** Angiography of the left main artery. **b** IVUS image of the left main stent at the ostia. **c** IVUS image of the sinus (the white dots indicate the proximal parts of the deformed stent). **d**, **e** Angiography and IVUS image after the second chimney stenting and postdilation. **f** Illustration of the position of the coronary stents in relation to the transcatheter heart valve

An emergency angiogram revealed severe stenosis of the ostium of the LM artery. Compared with the aortic angiography after TAVR, the stenosis of the LM artery had worsened, although a 4.0–15 mm stent had previously been deployed. A Judkins left 4.0 guiding catheter was used via femoral access, and a standard 0.014-inch guidewire was floated into the coronary artery and delivered to the distal left circumflex artery. The ostium of the LM artery and the mesh of the THV were dilated by gradually increasing the balloon size. Intravascular ultrasound (IVUS) revealed that the stent stenosis was caused by compression of the THV (Additional file 1), and no thrombus or in-stent restenosis was observed (Fig. 3b, c). Finally, under the support of the Guidezilla extension catheter (Boston Scientific, Boston, MA), a new 4.0–15 mm integrity stent (Medtronic, Santa Rosa, CA) was deployed. After the second chimney stent, the severe stenosis of the ostium of the LM artery disappeared (Fig. 3d), the IVUS showed this in more detail (Additional file 2).

## Discussion and conclusions

Chimney stenting is an effective method for coronary protection during the TAVR procedure [4]. Our case is the first to show that for self-expanding valves, even if the coronary ostium is protected by chimney stenting, with no severe stenosis immediately after the TAVR procedure, there is still a risk of delayed CAO, which can lead to cardiovascular events.

This patient had severe stenosis of the LM artery; thus, one stent was implanted with sufficient length to extend into the aorta before the TAVR procedure. It is not a classic chimney stent for coronary stent implantation before the TAVR procedure, but it meets the definition of chimney stenting and the deployment of a coronary stent extending from the proximal portion of a coronary artery cranially, exteriorly, and parallel to the THV [4]. Only 4 months later, the coronary ostium developed severe stenosis; thus, we speculated that the coronary stent after the TAVR procedure should have been mildly squeezed and that the degree of stenosis was not serious at that time because she was asymptomatic, with the aortic angiography demonstrating good perfusion. However, as time passed, the diameter of the self-expanding valve tended to expand further, which eventually led to severe stenosis of the chimney stent.

Since the Venus-A self-expanding valve has a waisted shape, there may be two main reasons why the chimney stent of the RCA was not affected by the THV. Firstly, the RCA ostium was higher than the LM ostium (13.2 mm vs. 9.0 mm) and secondly, the size of the right sinus of Valsalva was larger than the left sinus of Valsalva (24.3 mm vs. 23.6 mm).

From the international Chimney Registry, 60 patients underwent chimney stenting among 12,800 TAVR procedures (0.5%), and 2 patients developed stent failure for in-stent restenosis and stent thrombosis during follow-up [4]. From our case, another reason that may affect the prognosis is delayed CAO because of the progressive compression of the coronary stent. In their study, it was mentioned that incomplete stent expansion was frequently encountered and necessitated placement of a second stent inside the previously sited stent, which means that metallic stents may have less radial strength to resist valve pressure.

As Harhash et al. [5] mentioned, percutaneous coronary intervention after TAVR is quite difficult and dangerous. Stenting in a previous stent is much more challenging because it is quite difficult for the guidewire to go through the THV mesh above the level of valve leaflets and then through struts of the chimney stent because the guidewire cannot pass through the ostium of the previous stent. In our case, after dilating the coronary stent structures and the valve mesh, the new chimney stenting restored coronary artery reperfusion. Here are potential complications and possible therapy choices of chimney stenting after the TAVR procedure in our knowledge (Table 1). Data on the management of antithrombotic therapy after TAVR and chimney stenting are scant, in the absence of other oral anticoagulation indications, dual antiplatelet therapy (DAPT) is recommended for at least 6 months like complicated percutaneous coronary intervention procedure, and further extension of DAPT duration may be helpful.

**Table 1:** Potential complications and therapy choice of chimney stenting after the TAVR procedure

Potential complications	Occurrence time	Therapy choice
Stent deformation	Early, late	New chimney stenting, CABG
Stent thrombosis	Early, late	Thrombus aspiration, Balloon angioplasty, new chimney stenting, CABG
In stent restenosis	Late	Balloon angioplasty, CABG

To avoid this potentially serious complication, delayed CAO, careful selective angiography should be performed after the TAVR procedure, and clinicians should have a low threshold for performing coronary angiography when clinically suspected. New chimney stenting may be a bailout treatment for this situation.

## Supplementary Information

**Additional file 1**. The IVUS from LM stent to the aorta after a small balloon dilation.

**Additional file 2**. The IVUS after the second chimney stent deployment.

## Data Availability

All relevant data supporting the conclusions of this article are included within the article.

## Abbreviations

CAO: Coronary artery occlusion
DAPT: Dual antiplatelet therapy
IVUS: Intravascular ultrasound
LM: Left main
RCA: Right coronary artery
TAVR: Transcatheter aortic valve replacement
THV: Transcatheter heart valve
